# Manufacturing of Graphene-Nanoplatelet- and Carbon-Nanofiber-Filled PLA Composite Filaments for Tissue Engineering

**DOI:** 10.3390/polym18091058

**Published:** 2026-04-27

**Authors:** Eva Schätzlein, Phil Joel Groenewold, Salomé Luís, Annabelle Neuhäusler, Katrin Markus, Jannik Hallstein, Michael Großhauser, Yu Shrike Zhang, Andreas Blaeser

**Affiliations:** 1 Institute for BioMedical Printing Technologies, Technical University of Darmstadt, 64289 Darmstadt, Germany; schaetzlein@idd.tu-darmstadt.de (E.S.);; 2 Fraunhofer Institute for Structural Durability and System Reliability, LBF (Structural Durability and System Reliability), 64289 Darmstadt, Germany; 3 Division of Engineering in Medicine, Department of Medicine, Brigham and Women’s Hospital, Harvard Medical School, Cambridge, MA 02139, USA; 4 Harvard Stem Cell Institute, Harvard University, Cambridge, MA 02138, USA; 5 Broad Institute of MIT and Harvard, Cambridge, MA 02142, USA; 6 Centre for Synthetic Biology, Technical University of Darmstadt, 64289 Darmstadt, Germany

**Keywords:** FDM, FFF, muscle tissue engineering, electrically conductive PLA, C2C12, myoblasts

## Abstract

Electrical stimulation enhances functionality and accelerates maturation in biofabricated tissues, which are particularly important for muscle tissue engineering applications. Accordingly, there is demand for 3D-printable electrically conductive cytocompatible scaffolds that enable patient-specific geometries and localized electrical stimulation, as well as incorporate further maturation-promoting geometrical cues. Filament-based scaffolds from fused filament fabrication could overcome current limitations in geometric freedom, size and partially cytotoxic additives. In this study, biodegradable polylactic acid (PLA)-based conductive filaments incorporating graphene nanoplatelets (GNPs) or carbon nanofibers (CNFs) were developed via melt-mixing extrusion to possibly enable the electrical functionalization of muscle scaffolds. A two-stage process combining twin-screw and single-screw extrusion was preferred to allow for higher filler incorporation. Filament morphology, printability, electrical conductivity, and cytocompatibility were systematically evaluated. Homogeneous filaments containing up to 16 wt.% GNPs or 3.6 wt.% CNFs were successfully produced and processed by fused filament fabrication into scaffold geometries supporting myoblast orientation. Electrical conductivity was measured above 16 wt.% GNPs, with up to 2.7 µS/m, with printed constructs capable of connecting a circuit. GNP-based filaments were cytocompatible, supporting myoblast attachment and elongated morphology. An adjustable electrical stimulation setup demonstrated improved muscle maturation and contractile responses of C2C12 myoblasts, highlighting biodegradable conductive filaments’ potential for electrically active muscle tissue scaffolds.

## 1. Introduction

To advance muscle tissue engineering towards application in implants in regenerative medicine, researchers are aiming at enhancing construct functionality by increasing the cell fusion rate and contractility, promoting a more efficient tissue maturation and reducing the maturation time [1,2]. This can be achieved by applying specific cues present in native tissue development to design spatial–temporal-defined constructs for multi-tissue interface areas that present bone, muscle, or nerves, triggering muscle contractions in implants or biorobotic systems.

In particular, the development of mature myotubes has been shown to be positively influenced by topographical cues and electrical stimulation, improving the alignment, differentiation, metabolic activity and protein synthesis [3,4,5,6]. Microgrooves of 200 µm were shown to increase myotube length, myosin heavy chain content and maturation index in parallel-patterned scaffolds compared with non-structured counterparts [7]. Myotube-sized grooves (50 µm) have also been studied for cell alignment [3], and muscle maturation has been analyzed as a function of applied voltage (3–4.5 V) [8], with the setup shown to positively affect cell fusion efficiency. For example, Ahadian et al. were able to improve muscle tissue maturation using an interdigitated array of platinum electrodes [9] to apply electrical stimulation to C2C12 myoblasts, showing that their design was superior to the conventional two-wire setup commonly used to generate the electric field. Basurto et al. used a poly-pyrrole doped collagen scaffold for volumetric muscle loss treatment in mice [10,11]. Approaches using 3D printing for electrical stimulation of tissues show a higher degree of geometric freedom. Bilge et al. 3D-printed dissolved algae-based carbon and polycaprolactone (PCL) composites to create a porous scaffold with 800 µm of wire spacing [12]. However, current approaches lack applicability in regenerative medicine, as the lab-grown tissues are either limited in size, or in 2D cases, they are labor-intensive, use non-bioresorbable or partially cytotoxic materials, and have limited geometric freedom [10,11,12,13]. Zou and Vijayavenkataraman provided a recent overview of the currently used methods and materials for conductive materials in tissue engineering [14].

3D-printed multimaterial scaffolds offer the possibility to combine the advantages of the abovementioned approaches and create tissue engineered implants capable of regenerating large tissue defects, as they have a high degree of geometric and material freedom to support the formation of different tissues in the same construct. For example, an electrically conductive material can be printed to form microgrooves [15] to support muscle cell development and a more flexible one can be used to recreate ligaments, generating large constructs with multiple tissue functions [16]. Also, 3D-printed multimaterial scaffolds allow for a large part of the stimulation setup to be integrated into the printed structure and the usage of bioresorbable materials eliminates the need for further manual processing steps, such as the removal of electrodes.

Suitable electrically conductive 3D-printable materials include conductive polymers such as polyaniline [17] or poly-pyrrole [13,18,19,20], which can only be printed as a monomer and crosslinked via cytotoxic radicalization with light-based techniques, such as stereolithography. Another option is combining poorly conductive polymers, such as polylactic acid (PLA), with conductive fillers in a melt-mixing process to create a filament that is printable via fused filament fabrication for electrical stimulation [21]. Here, metallic conductive fillers, such as copper or gold, are usually not used due to their cytotoxicity or high cost. Instead, carbon-based additives [12,22,23] are a favorable cytocompatible alternative [24,25]. In particular, graphene films, as well as graphene oxide particles in hydrogels, have been shown to induce spontaneous myogenic differentiation without additional chemical cues by enhancing the fibronectin and albumin adsorption and upregulating intercellular signaling, making it and its derivates like graphene nanoplatelets (GNPs) or carbon nanofibers (CNFs) promising fillers for conductive filaments for tissue engineering [26,27,28].

In the past, the focus of investigation for the fabrication of conductive composites and filaments was on single-polymer–single-additive combinations for example graphene nanoplatelets, carbon fibers, graphene, carbon nanotubes in a matrix of polyamide, polyethylene, PLA, polypropylene or polybutylene terephthalate [19,29,30]. Meanwhile, recent works shifted their focus on a combination of different fillers and or matrix polymers to improve conductivity or processability [19,29].

By a combination of fillers with different geometries, the probability of the particles touching and forming a conductive pathway in the non-conductive matrix can be increased without increasing the overall filler content [31,32]. Another method of increasing the conductivity and processability is the mixing of matrix polymers introducing compartments with locally higher filler content that form conductive pathways with a second matrix polymer [22,23,33].

At the same time the overall viscosity of the molten polymer can be decreased with the additional matrix polymer, which can be favorable due to the fillers tendency to increase the viscosity of the molten polymer matrix, therefore also improving the processability [22,23,33,34].

Melt mixing or solvent casting are often employed, where the latter often achieves higher filler contents of up to 25 wt.% with a broad range of achieved electrical properties from 10^−8^ to 10^1^ S/cm [20,35]. Recent overviews of conductive filaments are included in the works of Misiak et al. [29] and Ryan et al. [19]. The previous works are promising regarding printability and conductivity; however, they are missing design considerations regarding applications in tissue engineering, like biodegradability or analysis of cytocompatibility.

For instance, compared with solvent-based composites, melt-mixed composites do not contain cytotoxic solvent residues that are favorable for tissue engineering applications. However, they require more knowledge for homogenization due to the higher viscosity of the matrix polymers, as the particle distribution in the melt-mixing process is mainly influenced by the surface energy of the filler and matrix, viscosity, particle diffusion, and the parameters of the blending [23,31].

Commercially available conductive filaments used for 3D printing via fused filament fabrication usually use non-degradable base material and additives such as plasticizers like phthalates, lubricants, and thermal stabilizers to improve manufacturing and printability [36,37]. These additives can negatively affect the viability and biofunctionality of cultured cells [38,39]. Thus, there is a need for the development of electrically conductive filaments without toxic additives so that they can be applied in fabricating scaffolds for improvement of muscle maturation and later tissue implantation.

This work analyzes the development of an electrically conductive and cytocompatible filament, with a particular focus on the properties relevant to muscle tissue engineering. For this, electrically conductive fillers—GNPs or CNFs—were embedded in a biodegradable PLA matrix in a melt-mixing process and compared regarding the final filament morphology, 3D-printability, electrical conductivity and biological compatibility.

## 2. Materials and Methods

### 2.1. Composite and Filament Production

In our previous work, we established a process to manufacture composite filaments with up to 20 wt.% filler content in micrometer size using a single step process with a desktop single-screw extruder (NEXT 1.0 Advanced, 3devo B.B., Utrecht, The Netherlands) [15]. In this work, we compare this extruding procedure with a procedure that includes an additional compounding process in a twin-screw extruder for the production of filaments with particles in nanometer size.

Before compounding, Luminy L175 (TotalEnergies Corbion, Gorinchem, The Netherlands) PLA pellets were milled (type 1528, Rapid Granulier Systeme GmbH & Co.KG, Kleinostheim, Germany) and dried for at least 12 h at 75 °C in a vacuum oven. Mixtures of 15 wt.% graphene nanoplatelets (900407, <2 μm particle size, Sigma-Aldrich, Taufkirchen, Germany), as well as 5 wt.% carbon nanofibers (719811, 100 nm × 20–200 µm, Sigma-Aldrich, Taufkirchen, Germany) with milled PLA, were prepared and mixed manually. This premix was then added into the feeder of the co-rotating twin-screw extruder (Thermo Scientific Process 11 Twin-screw Extruder, Thermo Fisher Scientific, Karlsruhe, Germany), which automatically dosed the mixture into the extruder.

**Figure A1:**
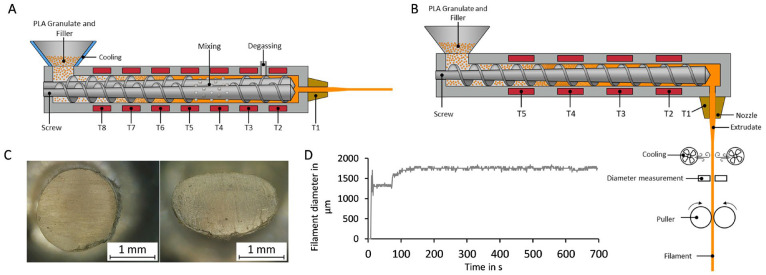
Filament production process. (**A**) Scheme of the twin-screw extruder with the different heating zones. (**B**) Scheme of the single-screw extruder with the different heating zones. (**C**) Images of cross-sections of filaments extruded at 165 °C and 190 °C nozzle temperatures of the single-screw extruder. (**D**) In-line measurement of filament diameter during extrusion of 1% GNPs in the single-screw extruder using the two-stage process.

The twin-screw extruder has seven heating zones and a temperature control for the nozzle (T1–T8, Figure A1). The exact extrusion parameters for the various mixtures are shown in the following table (Table 1).

The extrudate was drawn through a water bath (567-7670, Thermo Fisher Scientific, Karlsruhe, Germany) into a pelletizer (567-7672 VariCut Pelletizer, Thermo Fisher Scientific, Karlsruhe, Germany) and cut into pellets of approximately 1.5 mm.

For the filament production, mixtures with 1, 2, 3, 4, 5, 7, 11, 15 and 20 wt.% GNP; mixtures with 1, 2, 3, 4 and 5 wt.% CNF with PLA granules; and pre-compounded pellets with PLA granules were prepared using a desktop single-screw extruder NEXT 1.0 Advanced, 3devo B.B., Utrecht, The Netherlands). The exact extrusion parameters for the various mixtures are shown in the following table (Table 2, Figure A1).

To achieve the desired filament diameter of 1.75 mm, the speed of the conveying mechanism was set to automatic.

Prior to the extrusion process, the single-screw extruder was cleaned by extrusion of 12 g cleaning pellets (Dyna-Purge, Shuman Plastics Inc., Buffalo, NY, USA) for roughly 5 min and a sequential extrusion of PLA pellets for 20 min to flush out previous extruded polymers. The filament extrusion was started with PLA pellets to allow for the pulling mechanism to stabilize and produce filament in the desired filament diameter. Then, the PLA-filler mixtures were added to the now empty hopper of the single-screw extruder, and the first 25 g of the composite filament were discarded to avoid analyzing a mixture of the desired composite with the remaining PLA pellets used for flushing.

### 2.2. Filament Characterizations

The composite filaments made in the single- and two-stage processes, as well as three commercial filaments, were analyzed. For the latter, commercial filament 1 (C1) (CP90039-1-S 3dkonductive 2022, 3dk.berlin, Berlin, Germany), commercial filament 2 (C2) (CDP1170 Electrically Conductive Composite PLA, Protoplant INC, Vancouver, WA, USA) and commercial filament 3 (C3) (Electrifi Conductive Filament, Multi3D, Middlesex, NC, USA) were chosen at random.

Scanning electron microscopy (SEM) energy-dispersive X-ray spectroscopy (EDX): Using the scanning electron microscope (Zeiss EVO 10—SmartSEM touch, Carl-Zeiss AG, Jena, Germany) tensile fractures, cryo-fracture cross sections of the filaments and fracture surfaces of printed samples were analyzed. EDX images were taken with the software (Zeiss SmartEDX, Carl-Zeiss AG, Jena, Germany). The images were acquired in variable pressure mode with sputtering only for tensile fractured samples using the backscatter detector. Adjustable parameters were selected as described in Table 3.

Roughness measurement: The surface roughness of the filaments was analyzed with a 3D-Profilometer (VR-6000, KEYENCE Deutschland GmbH, Frankfurt am Main, Germany) using the micro function with 40× magnification and the high-resolution mode. Using the surface shape correction function, the waviness was removed at a cut-off size of 0.5 mm. Furthermore, an S-filter of 10 µm was applied. For each filament, the surface roughness was analyzed in four areas (6 mm × 2 mm × 1 mm) (N = 1). The line roughness was analyzed in multiple areas of the filament in direction relative to the filament, as well as perpendicular to the filament direction with a length of approx. 1.5 mm (N = 1, n = 32).

Cross-section analysis: Continuous measurements of the filament cross-section during the extrusion process were made with the integrated sensor of the single-screw extruder. Furthermore, the filament diameter was measured on a random 100 cm-long piece using a digital caliper (WZ0031, LogiLink, Schalksmühle, Germany). The measurements were taken at intervals of 10 cm (N = 11). At each point, two measurements were taken with an axial rotation of 90° to each other in order to determine possible ovality. For this purpose, the average diameter $\bar{d}$ resulting from the two measuring axes was first calculated and then the deviation in percent $d_{o}$ from the smaller value of the two measuring axes was determined:

(1) $$d_{o}\;=\frac{100}{\bar{d}}\;\cdot \;d_{min}$$

- $d_{o}$: Deviation of the diameter from a perfect circle in %;
- $\bar{d}$: Average diameter;
- $d_{min}$: Minimum diameter of the measuring point.

Tensile testing: The mechanical properties of the filaments were determined using a tensile testing machine (Z050, ZwickRoell, Ulm, Germany) (N = 4). The environmental conditions were kept stable at 23 °C and 50% humidity according to DIN EN 20187. Specimens of 30 mm length were mounted using a 1 kN load cell, an initial load of 1 N, and a constant deformation speed of 10 mm/s according to DIN 53455.

Thermogravimetric analysis: To determine the filler content of the compounds and filaments, a thermogravimetric analysis (Mettler Toledo, Columbus, OH, USA) was executed and analyzed with STAR^e^ SW software (Version 16.0, Mettler Toledo, Columbus, OH, USA) (N = 1)). Measurements were done in a N_2_ atmosphere with a heating rate of 10 K/min, start temperature of 25 °C, end temperature of 600 °C and measurement interval of 1 s.

### 2.3. Printing and Analysis of Printability

CAD and slicing software: The CAD program Autodesk Inventor (Professional 2020 Build 400, San Francisco, CA, USA) was used to design the 3D-printed components in this project. The components were then exported as STL files and prepared for 3D printing using the PrusaSlicer slicing program (version 2.3.3+win64, Prusa Research a.s., Prague, Czech Republic).

3D printing: To produce the test specimens for measuring the electrical properties and the models for printability analysis, the fused filament fabrication 3D printer model i3 MK3S (Prusa Research a.s., Prague, Czech Republic) equipped with a V6 stainless steel nozzle with a diameter of 0.4 mm (E3D-Online, Oxfordshire, UK) and a satin powder-coated sheet (Prusa Research a.s., Prague, Czech Republic) for improved component adhesion was used. This 3D printer is designed for filaments with a diameter of 1.75 mm. The material-dependent printing temperature was determined for each filament. For this purpose, a sample printing geometry was printed from 200 °C to 280 °C and a suitable printing temperature was selected for each filament to analyze the stringing, shape fidelity and print resolution (N = 1). Using the same geometry and the best printing temperature, the smallest positive and negative printed features were analyzed (N = 1).

### 2.4. Electrical Characterizations

Analysis of conductivity: To measure the specific electrical resistance of the composite filaments using four-point contact measurement, test specimens were 3D-printed in accordance with DIN EN ISO 3915. The test specimens were 10 mm wide, 80 mm long, and 3 mm thick. The printing direction was in the shortest direction between the measurement electrodes. The specific electrical resistance or electrical conductance could be determined using the geometry of the test specimens and Ohm’s law (N = 4). A series 2400 Source meter (Keithley Instruments, OH, USA) was used as the current source. The voltage was measured using a digital multimeter (Voltcraft VC250, Conrad Electronic SE, Hirschau, Germany). The measurements were taken at least 12 h after the test specimens were produced by the 3D printer. Before the test, the test specimens were stored for 120 min in the measurement environment at 23.5 °C and a relative humidity of 41% so that each test specimen could reach the same temperature.

Analysis of surface temperature of printed samples under electrical load: A printed sample of 20 wt.% (calc.) GNPs with a size of 30 mm × 3 mm × 1 mm was covered with silver ink (Loctite ECI 1010, Henkel Electronic Materials Westerlo, Westerlo, Belgium) on the contacting points and a voltage of 16 V was applied by a source meter (Konstanter SLP 240-80, Gossen Metrawatt, Nuernberg, Germany) for 13 min. The surface temperature of the sample under load and a control sample without the applied voltage were measured at the same time using a thermal imaging camera (FlirOne Pro, Frankfurt am Main, Germany) (N = 1).

### 2.5. Biological Characterization

Cell culture: A mouse myoblast cell line (C2C12, CLS, Heidelberg, Germany) at a passage below 40 was cultured in cell culture flasks in growth media in Dulbecco’s Modified Eagle’s Medium high glucose (Gibco, Life Technologies Limited, Parsley, UK) with 20 vol.% FBS (Thermo Fisher Scientific Inc. Waltham, MA, USA) and 1 vol.% Penicillin/Streptavidin (Gibco, Life Technologies Limited, Parsley, UK) until about 80% confluency and then passaged every 2–3 days at a 1:15 ratio. To initiate differentiation, a differentiation media was used, which contained Dulbecco’s Modified Eagle’s Medium, 2 vol.% horse serum (H1270, Sigma-Aldrich, Taufkirchen, Germany), and 1 vol.% Penicillin/Streptavidin. Cells were removed from the flask by washing with phosphate-buffered saline (PBS) (RotiCell 10× PBS, Carl Roth GmbH+ Co. KG, Karlsruhe, Germany), trypsination (TrypLE™ Express Enzym, Thermo Fisher Scientific Inc., Waltham, MA, USA) for 5 min and resuspension in growth media. For experiments involving differentiation of cells, the cells were cultured in a cell culture incubator (37 °C, 5% CO_2_, CO_2_ incubator, Binder, Tuttlingen, Germany) at confluency for two days in growth media prior to harvesting to induce differentiation. To assess the contractibility, cells were cultured in differentiation media for three weeks after cell seeding in an 8-well plate (µ-Slide 8 Well high, ibidi GmbH, Gräfelfing, Germany) (N = 3). To analyze the effect of electrical stimulation on differentiation, cells were cultured in differentiation media for 4 days until early myotubes appeared and were analyzed after continuous electrical stimulation for an additional 4 days, with the pulsed signal at 3 V/cm (N = 3).

Measurement of cytocompatibility: The cytocompatibility of the filaments was assessed by determining the metabolic activity of the C2C12 myoblasts in response to the conditioned media using a Celltiter blue (CTB) assay (Promega Corporation, Fitchburg, MA, USA) (N = 3). Briefly, 2 cm long filaments were sterilized in 70% ethanol, followed by washing in sterile PBS and immersed for 24 h in 1.5 mL growth media at 37 °C. The preconditioned media was then applied to 10^4^ cells/cm^2^ seeded in a 96-well plate (VWR Tissue culture plate, 734-2781, VWR International LLC, Radnor, PA, USA) one day prior and incubated at 37 °C for 24 h in 5% CO_2_. The media was removed and a CTB solution of 100 µL of growth media with 20 µL of CTB solution (Promega Corporation, Fitchburg, USA) was added to each well and incubated at 37 °C for 2 h. The fluorescence intensity of 100 µL of the supernatant was read with a plate reader (Infinite M Plex, Tecan Group AG, Männedorf, Switzerland).

Analysis of the cell morphology and differentiation: Printed samples were sterilized in 70% ethanol followed by washing in sterile PBS, and C2C12 were seeded on the samples at a concentration of 10^5^ cells/cm^2^. After one day of incubation at 37 °C for 24 h in 5% CO_2_, the samples were transferred to a new well plate and stained with a live–dead stain (L3224, Thermo-Fisher Scientific, Waltham, MA, USA) and analyzed via fluorescence microscopy (Zeiss Axio Observer Z1, Carl Zeiss AG, Oberkochem, Germany) (N = 1).

Assessment of the cell differentiation was done by immunofluorescence staining (N = 3). For this, cells were fixed in 4% paraformaldehyde (Carl Roth GmbH + Co. KG, Karlsruhe, Germany) for 1 h and permeabilized with 0.5% Triton X-100 in PBS (Carl Roth GmbH + Co. KG, Karlsruhe, Germany) for 30 min. For staining myosin heavy chain, samples were first incubated with 5 vol.% goat serum (S26-M, Sigma-Aldrich, Taufkirchen, Germany) for 30 min, washed with PBS and then sarcomeric *α*-actinin was labeled with the primary antibody (Monoclonal Antibody EA-53, Invitrogen Thermo-Fisher Scientific, Waltham, MA, USA) (1:500 dilution in PBS). After incubation overnight, the samples were stained with the secondary antibody AlexaFluor594 goat anti-mouse (A11005, Invitrogen, Thermo Fisher Scientific Inc. Waltham, MA, USA) for 30 min (1:500 dilution in PBS). Actin filaments were stained with Alexa Fluor 488 Phalloidin (1:400 dilution in PBS) (A12379, Invitrogen, Thermo Fisher Scientific Inc. Waltham, MA, USA) and nuclei for 30 min with 4′,6-diamidin-2-phenylindol (DAPI) (1:800 dilution in PBS) (MBD0015, Sigma Aldrich, St. Louis, MO, USA). Microscopic images were taken by bright-field and fluorescence microscopy with a light microscope (Echo Revolve, Discover Echo Inc. San Diego, CA, USA) and a confocal microscope (TCS SP8 Leica Microsystems, Mannheim, Germany). The morphology of differentiated cells was analyzed by highlighting the cells and using the analyze particles function in Image J (ImageJ 1.54f, National Institutes of Health, Bethesda, MD, USA). For the myotube length analysis, for each condition, more than 318 cells were counted, and for the analysis of nuclei/myotube, more than 54 cells were analyzed for each condition.

### 2.6. Electrical Stimulation Setup

A microcontroller STM32 Nucleo-64 (L476RG, STMicroelectronics N.V., Plan-les-Ouates, Switzerland) was programmed with the Arduino IDE (Version 2.3.6, Arduino SA, Lugano, Switzerland). Using the analog outputs D13 and A2, 2 ms short positive pulses were generated one after the other, with a pause of 196 ms to 496 ms. The signals on each output had an unwanted offset (around 0.1 V) and noise. Each signal was amplified 2–10× in a closed loop amplifier (MCP604, Microchip Technologies INC., Chandler, AZ, USA). The power source of the microchip was 5 V, and the ground of the STM32 was additionally connected with two capacitors (220 pF, 1000 µF) to lower the noise of the generated signals. The two amplified signals were connected with a capacitor (220 pF) to lower the noise and to the electrodes in contact with the cells.

Using a custom-designed printed circuit board allowed for parallel stimulation of up to 8 samples and imaging during simulation. Electrodes from graphite foil (Graphite Foil, ProGraphite GmbH, Untergrießbach, Germany) and printed filaments in the size of 1 cm × 1 cm × 0.5 mm were attached with small clamps. The setup was used with graphite electrodes at 1 V and 0.08 mA and at 2 V and 0.16 mA, as well with printed 20% GNP electrodes at 5 V and 0.1 mA.

### 2.7. Statistical Analysis

Statistical significance was determined with a two-sample *t*-test, one-way ANOVA and Tukey’s HSD. Graphical data shows the mean with error bars as the standard deviation with significance depicted as n.s. for no significance and * for *p* < 0.05, with α = 0.05.

## 3. Results and Discussion

The aim of this study was to analyze the production of an electrically conductive and cytocompatible filament for muscle tissue engineering to improve tissue regeneration in multi-tissue interface areas.

Based on our previous work [15], a filament made of PLA, a material already well established in TE, was functionalized by embedding electrically conductive fillers, i.e., GNPs or CNFs, into the matrix polymer using a melt-mixing process without any additional additives.

### 3.1. Filament Production

GNPs and CNFs were selected for the electrical functionalization of PLA, as they offer excellent conductivity due to their high surface-to-volume ratio, which is advantageous for the formation of a conductive path through the non-conducting matrix to produce a percolation network [40,41]. GNPs are resistant to corrosion, have a high biocompatibility [24,25], and have a positive influence on myogenic differentiation due to the induction of spontaneous myogenic differentiation [26,27,28]. CNFs have rarely been studied in the context of filament production and medical applications, and their main difference from GNPs is their higher aspect ratio (length to width) [31]. The long, thin fibers increase the probability of contact between the fillers, and could thus can improve the formation of the percolation network [32,41].

**Figure 1:**
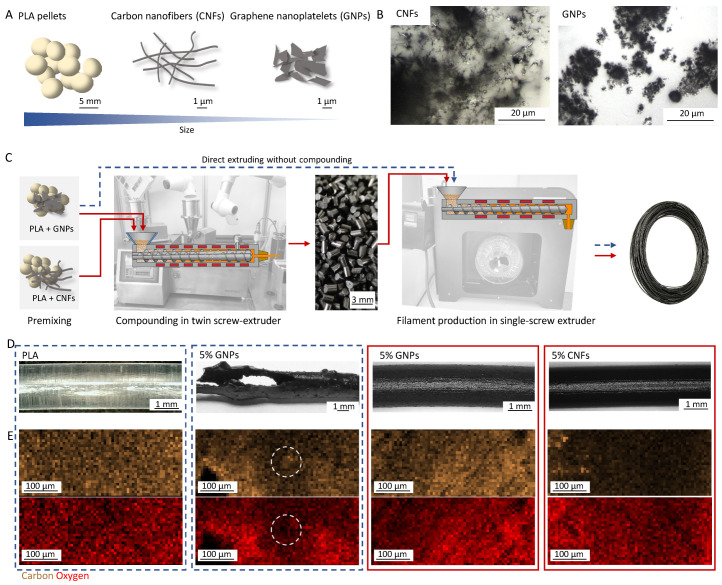
PLA embedded with electrically conductive fillers filament production. (**A**) Size comparison of the matrix material (PLA pellets) and the conductive fillers (CNFs and GNPs). (**B**) Microscopic images of CNFs and GNPs agglomerates. (**C**) Filament production processes: blue—single-stage process using a single-screw extruder to extrude filaments with up to 5 wt.% (calc.) GNPs; red—two-stage process using an additional compounding step in a twin-screw extruder for production of pellets with 0 wt.% and 5 wt.% (calc.) CNFs and 15 wt.% (calc.) or 20 wt.% (calc.) GNPs. (**D**) Resulting filaments with filler content of 5 wt.% (calc.) GNPs made in the single- (blue) and two-stage (red) processes and 5 wt.% (calc.) CNFs made in the two-stage (red) process. (**E**) SEM-EDX area scans of filament cross-sections with carbon (brown) and oxygen (red) visualized. A possible GNP agglomeration is highlighted in white.

Two different manufacturing processes were examined (Figure 1) in this study. The hygroscopic PLA pellets (Ø ≈ 5 mm) were first dried to enable uniform processing, as the moisture content would have a significant influence on the rheology of the polymer melt [42,43,44]. GNPs and CNFs are not considered hygroscopic [25,45] and were therefore not dried beforehand (Figure 1A,B).

After weighing the PLA pellets, they were manually mixed with the electrically conductive fillers, and the mixtures were either melted directly in a single-screw extruder and processed into a filament (single-stage process) or underwent an additional upstream compounding step in a twin-screw extruder (two-stage process) (Figure 1C). Filler concentrations are based on calculated values (calc.), taking into consideration the proportions PLA/filler present in the manual premix. The comparison of the single- and two-stage processes was done with GNP fillers. The highest possible extrudable amount of filament was with 5 wt.% (calc.) GNPs in the single-stage process, although the resulting filament was very inhomogeneous (Figure 1D), as large inclusions of unmixed GNPs were noticeable, and it also had a highly irregular diameter that deviated significantly from the target 1.75 mm, mainly due to the filament’s high waviness degree. In the two-stage process, the highest filler contents added to the polymer melt were 5 wt.% CNFs and 20 wt.% GNPs, as higher concentrations lead to inhomogeneous mixing or clogging of the twin-screw extruder. The filler distribution in the filament cross-section was analyzed via SEM-EDX using the elements carbon and oxygen (Figure 1E). PLA (chemical formula of the monomer: C_3_H_4_O_2_) consists of carbon, oxygen and hydrogen, while GNP and CNF are carbon-based. Therefore, measurement of filler agglomeration in the PLA matrix is difficult but can be estimated by a local decrease in the oxygen signal and a simultaneous carbon signal, which is increased depending on the filler concentration. The signal height is also influenced by the surface topography. Therefore, the analyzed cross-sections, which were made by freezing and then breaking, introduce errors in the signal for instance drops in the signal due to an uneven fracture surface, which have to be considered when analyzing the filler distribution in the filament.

The overall ratio *r_oc_* of oxygen to carbon atoms in the area scan of the PLA sample was 0.35, which differs from the theoretical ratio *r_oc,theo_* of 0.67 for the PLA sample.

For samples with 5 wt.% fillers, *r_oc,theo_* is 0.63, and for the 15 wt.% filler, *r_oc,theo_* is 0.5. Fillers without the matrix have an *r_oc,theo_* of 0. The measured *r_oc_* values in the area scans are 0.39 for 5 wt.% CNF and 0.41 for 5 wt.% GNP in the one- and two-stage processes, and 0.37 for 15 wt.% GNP and 0.04 for the 100% GNP sample. The measured *r_oc_* values vary a lot from the theoretical values; therefore, EDX data can only be considered for a rough estimation of particle agglomeration. A quantitative analysis of the filler content was conducted separately via a thermogravimetric analysis.

The area scan of the PLA filament cross-section reveals a carbon and oxygen signal with high variation. This variation is also visible in the area scans of the other samples (5 wt.% CNF, 5 wt.% GNP). Only the sample with 5 wt.% GNP made in the single-stage process shows slight hints of larger carbon agglomerates (>50 µm), with local drops in the oxygen signal with a simultaneous increasing carbon signal visible.

Therefore, considering the high error of the measurement, our analysis indicates a homogeneous distribution of the fillers in the PLA matrix, with no large agglomerates in the filaments made in the two-stage process.

Particle localization in the filament is determined by the several components’ surface energies, and therefore, their wetting and thermodynamic behavior, as well as other mixture-related kinetic factors, such as viscosity, temperature, duration of mixing, sequence of mixing, and shear rate of the extruders [22,31].

The calculation of the interfacial energies of PLA, GNPs and CNFs with the Owens–Wendt model (literature values: PLA—solid surface energy: 40 mJ/m^2^, dispersive component: 35 mJ/m^2^, polar component: 5 mJ/m^2^ [22]; GNPs—solid surface energy: 59 mJ/m^2^, dispersive component: 33 mJ/m^2^, polar component: 26 mJ/m^2^ [22]; CNFs—solid surface energy: 56 mJ/m^2^, dispersive component: 50 mJ/m^2^, polar component: 6 mJ/m^2^ [46]) indicates a slightly lower interfacial energy between PLA and CNFs compared with the interfacial energy between PLA and GNPs. This lower interfacial energy should lead to better miscibility. In our study, we could not confirm this, which aligns with other studies that found that the viscous properties/kinetic properties dominate over the effect of the components’ surface energies [22,31]. Comparing the maximum filler content in filaments produced using the two-stage process, i.e., with the twin-screw extruder, reveals that GNPs can be incorporated in a higher concentration than CNFs. This may be attributed to differences in the particle morphology. Specifically, the high aspect ratio of CNFs may require higher shear forces to achieve uniform dispersion with the PLA matrix [47,48]. The importance of shear forces is furthermore highlighted by the maximum GNPs content of filaments and compounds created in the twin- and single-screw extruders. By using the same material, its properties, and therefore the thermodynamic behavior, are the same in both processes, with the main difference being the shear forces produced by the machines, showing a dominating kinetic effect for filament mixing. Additionally, the temperature profile in the extruder plays a key role in mixing. As the temperature rises, the viscosity of the polymer melt decreases, as does the force required to mix the molten PLA with the chosen filler, allowing for a higher filler content [44,49]. However, the determined thermal decomposition temperature of the PLA in our study (300 °C) sets an upper limit for the processing temperature range [50]. Hence, another possibility to lower the viscosity of the polymer melt is to include other additives (e.g., plasticizers such as polymers with a lower melting point, such as PCL) to further increase the electrically conductive filler content [22].

The temperature of the heating zones should be set with a lower temperature in feeding zone to avoid polymer agglomerates in the hopper and further clogging issues (Figure A1A, B). Also, it is necessary to have the parts closer to the extruding nozzle at a reduced temperature (165 °C) to allow for deliberate filament diameter control, which is only possible with a higher viscous melt, and avoid an introduced oval filament shape by the pulling mechanism (Figure A1C).

To track the filament quality during the filament extrusion process in the single-screw extruder, its diameter was tracked with a built-in diameter measurement of the extruder in relation to the extruding time (Figure A1D). The resulting analysis showed variations from the set diameter of 1.75 mm (Figure 1C), with drops to zero usually being caused by filament detaching from the conveying mechanism, where it cannot be measured. The initial part of the process showed the highest variations, demonstrating the need for around 1.6 min to achieve a stable extruding process.

### 3.2. Physical Characterization of the Filaments

**Figure 2:**
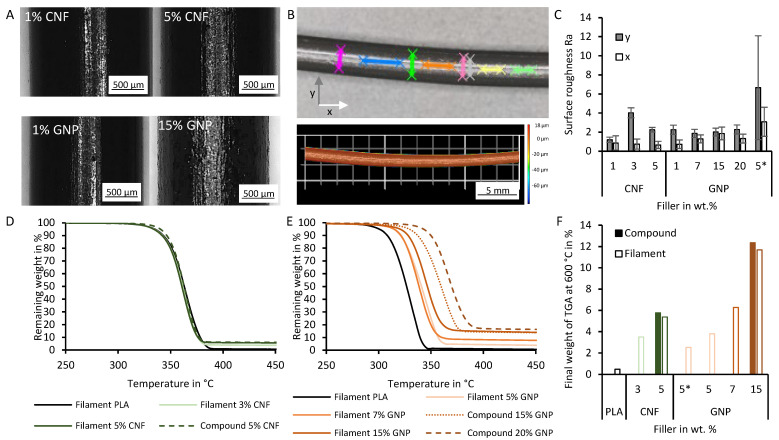
Physical characterization of the filaments. (**A**) Macroscopic images of filaments produced in a two-stage process. (**B**) Locations of roughness analysis and height map of the filament after filtering to remove the curvature of the filament. (**C**) Line surface roughness of the filaments produced in the two-stage process; the sample marked with * was produced in the single-step process. (**D**) Thermogravimetric analysis of compound from the twin-screw extruder and the filaments made in the two-stage process of CNFs. (**E**) Thermogravimetric analysis of compound from the twin-screw extruder and the filaments made in the two-stage process (7 wt.% (calc.)) and the single-stage process (5 wt.% (calc.)) using GNPs. (**F**) Final weight of the TGA of materials made in the two-stage process; the sample marked with * was made in the single-stage process.

**Figure A2:**
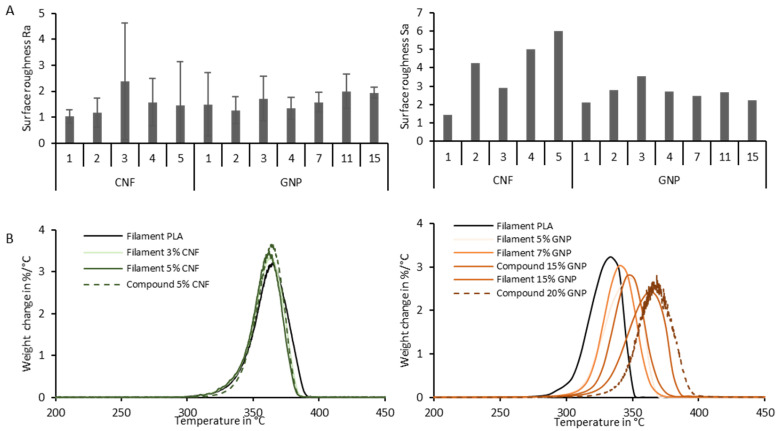
(**A**) Surface roughness measurements of composite filaments made in the two-stage process. (**B**) Derative thermogravimetric analysis of CNF and GNP compounds and filaments.

The filaments obtained by the two-stage process are optically distinctive when the filler content is increased for both GNPs and CNFs (Figure 2A). As the filler proportion increases, the filaments become less reflective, dispersing light more strongly. To quantify this effect, the filament surface roughness (Sa) was determined (Figure 2B, C), but showed no significant differences (Figure A2A). When this analysis was broken down into line roughness in the direction (x-direction) and perpendicular (y-direction) to the filament, a visible trend emerges, despite the short measuring distances: in the GNP filaments, the line roughness (Ra) shows an increasing trend with increasing filler content when it is measured in the x-direction (Figure 2C). However, no clear trend is apparent in the y-direction, which can be attributed to an induced secondary roughness caused by the manufacturing process by the notches in the extruding nozzle by abrasion of the fillers [51]. Both effects are not statistically significant.

The TGA (Figure 2D–F) and the derivative TGA (DTGA) (Figure A2B) further detail the influence of the composition on the thermal properties of the filaments. With increasing GNP filler content, the start of thermal degradation begins at higher temperatures, which is visible as a drop in weight in the TGA during heating (Figure 2D,E) and as an increase in the DTGA. This behavior seems to be more prominent for the GNPs than the CNFs. The DTGA shows a later peak of the highest change in weight for the GNP composites. Furthermore, two thermal degradation mechanisms of PLA are visible with a flat slope for lower temperatures and a steeper slope at higher temperatures, which are described in the literature with a transesterification reaction and a free radical reaction respectively [52].

Other studies showed that the delayed mass loss of the polymer can be attributed to the filler’s ability to form a thermally stable network around the polymer matrix, which lowers the rate of heat transfer. The interfacial interaction of the PLA with the fillers could increase the required energy for polymer decomposition [22,53,54,55,56]. Furthermore, the radical scavenging of graphene could also lead to a delayed thermal degradation of the composites [22,57,58].

Looking at the final weight at 600 °C in the TGA of the different filaments, it is shown that the filler content is slightly lower than the calculated value from manual mixing of the PLA pellets and GNPs (Figure 2F). Also, it can be observed that the filaments have a lower measured filler content compared with their compound counterpart with the same filler content. This can be attributed to the small batch (approximately 50 g) produced for each filament since the single-screw extruder has residual PLA from the previous flushing and cleaning step. Furthermore, the GNP filament produced in the single-stage process has a much lower filler content than the starting mix, which can be attributed to its inhomogeneous mixing.

### 3.3. Filament Production

**Figure 3:**
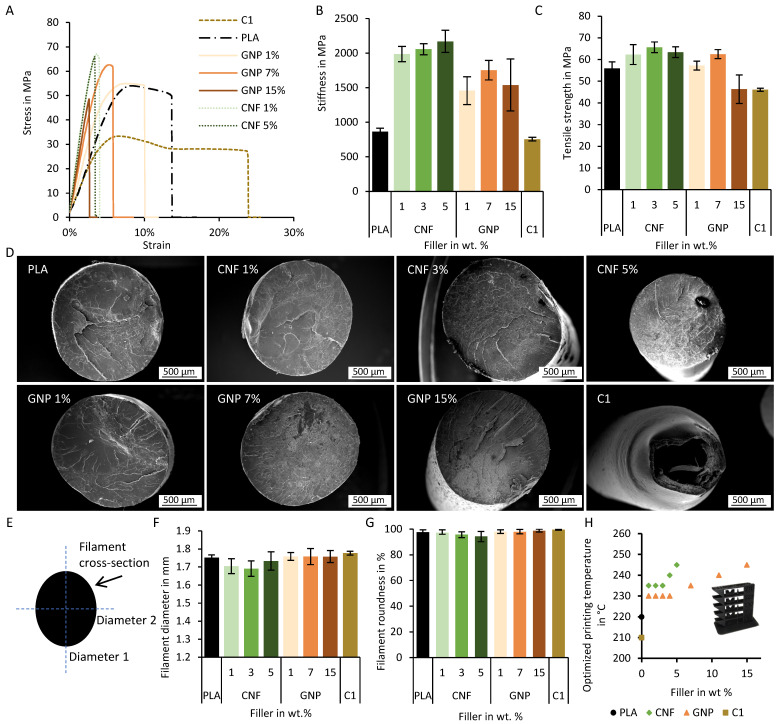
Mechanical characterization and printability of filaments made in the two-stage process. (**A**) Stress–strain curves. (**B**) Analysis of stiffness. (**C**) Analysis of tensile strength. (**D**) SEM images of fracture surfaces. (**E**) Scheme of a filament cross-section and measurement of filament diameter used for calculation of filament roundness. (**F**) Analysis of filament diameter. (**G**) Analysis of filament roundness. (**H**) Optimized printing temperature found by an analysis of print quality of a sample geometry.

Henceforth, all experiments were conducted with filament from the 2-stage process, as this possesses a higher filler content and is more homogeneously mixed. Mechanical characterization of the filaments using a tensile test was performed by analyzing the stress strain curve (Figure 3A), where the slope at the beginning of the curve from 0–3% strain is the stiffness; the maximal point is the tensile strength, and the tensile elongation at break showcases the ductility maximal strain until break.

Mechanical properties of the filaments depend on the filler geometry, percentage, orientation, dispersion, and adhesion [22,59]. Fillers and macromolecular polymeric chains can interact and form physical crosslinking points, and therefore, limit chain movement, resulting in an increased stiffness [22,60]. Also, particles can increase the crystallinity of the polymer, as they play a role in the heterogenous nucleation [22,53,60]. Our results show that the stiffness of the composite filaments is significantly higher than the control PLA filament (Figure 3B). Specifically, CNFs filaments showcase an increase in stiffness, even at low concentrations, compared with the samples with GNPs, highlighting the influence of the filler geometry in the final mechanical properties of the filaments.

Additionally, the tensile strength is slightly improved at lower concentrations (1–3 wt.% for CNFs and 1–7 wt.% GNP-containing filaments), but at higher concentrations, this property is lower compared with the PLA filaments (Figure 3C), showcasing two different effects. The interfacial adhesion between the fillers and the matrix can lead to an improved transference of stresses, and therefore, improve the final tensile strength of the filament. However, fillers also introduce inhomogeneities, which can act as starters for crack formation, thus lowering the tensile strength at higher concentrations [22,53,60]. Scanning electron microscopic images of the fracture surfaces show a clean fracture without ductile deformation and without a reduction in diameter, regardless of their GNP and CNF content (Figure 3D).

C1, a commercially available PLA-based filament with carbon black as the conductive filler [37], was also studied, showing a similar stiffness, a lower tensile strength, and a higher maximal strain than the control PLA filament. The tensile loading leads to a ductile deformation and lowered diameter, which can be seen in the scanning electron microscopic image (Figure 3D). This may suggest that additional additives like compatibilizers, coupling agents, lubricants, or stabilizers are included in the commercial filament to improve its processability and printability via fused filament fabrication, with the possible drawback of lower long-term biocompatibility, in addition to the possible genotoxicity due to the carbon black [47,48,61].

To utilize the full potential of conductive filaments in tissue engineering, the final scaffold should present topographical cues like microgrooves or other geometrical features, such as directed generation of an electrical field or hinges for micro-robotic systems. Hence, the material should be printable with fused filament fabrication. For this, the filament should fit through the smallest diameter in the printer. We used a Prusa i3 MK3 printer with a Poly-tetrafluoroethylene tube with an inner diameter of 2 mm. Furthermore, to achieve repeatable prints, as well as to avoid over- and underextrusion, the filament diameter and roundness should only slightly vary over the used length. The diameter (Figure 3E,F) of all analyzed filaments produced in the two-stage process are in an adequate range to allow for 3D-printing, which was defined as 1.75 mm ± 5%, ensuring a suitable grip by the feeding system [62]. The roundness of the filaments was calculated with Equation (1) (Figure 3G). The filaments’ deviation from the targeted diameter and roundness is higher than the commercially available filament C1. Hence, it was necessary to adjust the extrusion factor according to the measured filament diameter before printing to avoid over- or underextrusion.

Our 3D printer features a direct drive extruder that feeds the filament in the heating block, which allowed easy access and removal of the debris, as the CNF- or GNP-containing filaments tended to break due to their low ductility (Figure 3C).

Nozzle clogging due to fillers in the filaments was noticeable after prolonged printing times (>30 min). Generally, the nozzle-size-to-particle-size ratio should not be under 6.2 in order to avoid clogging [15,63,64,65]. Using a 0.4 mm nozzle and GNP fillers with a maximal size of 2 µm, the calculated ratio was 200, and therefore, the filament was considered printable. However, agglomerates can increase the filler size and cause further nozzle clogging [66,67,68]. For the used CNF particles, with a maximal size of 200 µm, the calculated ratio was 2, which explains the observed occasional nozzle clogging.

Sample geometries of the filaments printed at temperatures from 200 to 280 °C were analyzed and the optimal printing temperature was selected based on the visual print quality (stringing, shape fidelity, print resolution) (Figure 3H). With increasing filler content of the composite filaments, the optimal printing temperature also increased, with a maximal temperature of 245 °C for 15 wt.% (calc.) GNP and 5 wt.% (calc.) CNF. Even though the thermal conductivity increased with the added fillers [69,70,71], which should result in a lower preferred printing temperature, other dominating effects were also influencing the printing temperature. The included fillers could increase the crystallinity, influencing the mechanical properties, and also increase the melting temperature [22,53,60,72]. In addition, the TGA (Figure 2D,E) showed that the carbon-based fillers had a higher thermal capacity. This, in turn, resulted in a higher printing temperature being required as well [66].

**Figure A3:**
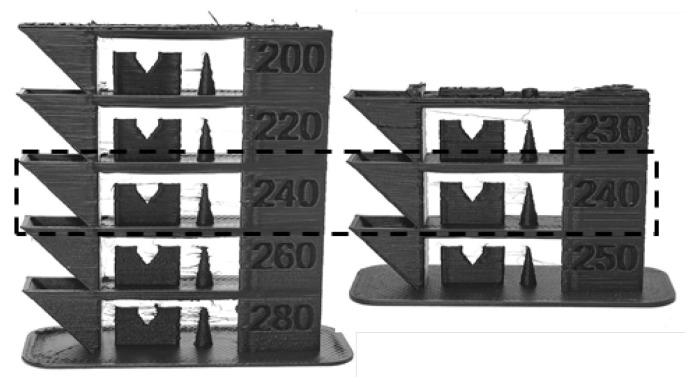
Sample geometries of 3% GNPs printed with a printing speed of 25 mm/s (**left**) and 60 mm/s (**right**). The printing temperature varied from 200 °C to 280 °C, as marked on the sample subunit.

To achieve a good printing quality, alongside the shape accuracy, the printing speed should also be taken into consideration. At higher printing speeds, a greater heat flow was necessary to melt the increased volume flow of the material. Due to the heat capacity and the thermal conductivity of the composite filaments, the lowest possible printing temperature at higher printing speeds (60 mm/s compared with 25 mm/s) was 230 °C (Figure A3).

Using all filaments made in the two-stage process, it was possible to print geometries that were successfully shown to aid cell alignment [3,7,8,9]. The smallest produced groves for 15 wt.% (calc.) GNPs were 390 µm and the smallest pillar was 545 µm in diameter. For 5 wt.% (calc.) CNFs, the smallest produced groove was 567 µm and the smallest pillar was 640 µm in diameter. These values are similar to the ones obtained with the commercial filament C1, with the smallest groove being 565 µm and the smallest pillar being 493 µm in diameter.

### 3.4. Electrical Characterization of the Filaments

**Figure 4:**
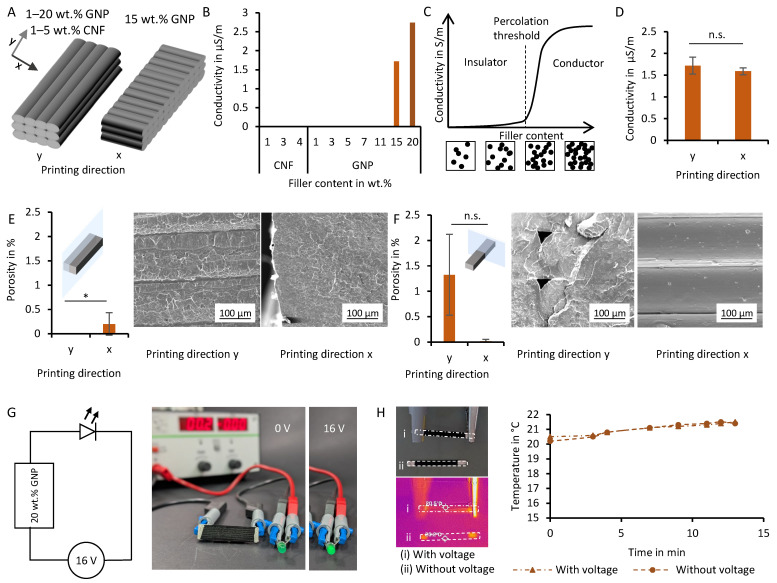
Electrical characterization of the composite filaments. (**A**) Schematic of the samples measured with and perpendicular to the printing direction. (**B**) Conductivity of samples printed along the measurement direction. (**C**) Visualization of conductivity and the influence of conductive filler content on the formation of a percolation threshold (adapted from [41]). (**D**) Conductivity of 15 wt.% (calc.) GNPs printed along and perpendicular to the measurement direction. (**E**) Porosity and SEM images of cross-sections along the measurement direction of the printed 15 wt.% samples. (**F**) Porosity and SEM images of cross-sections perpendicular to the measurement direction of the printed 15 wt.% samples. (**G**) Scheme and image of an electrical circuit illuminating an LED with an included printed sample from 20 wt.% (calc.) GNPs. (**H**) Measurement of surface temperature of printed sample of 20 wt.% (calc.) GNPs with an applied voltage of 16 V DC via thermal imaging and quantification. Statistical significance * for *p* < 0.05. n.s. for no statistical significance.

The conductive fillers GNPs and CNFs were added to achieve electrical functionalization of the PLA matrix. The electrical properties of the filaments produced in the two-stage process were analyzed using a four-probe measurement (Figure 4A, B, D). For this, 3D-printed samples with a printing orientation in the direction of the measurement were analyzed, as the printing direction can introduce cavities, impacting the conductivity [22,73]. Insulating materials, such as PTFE and PLA exhibit very low conductivity, around 10^−24^ S/m and 10^−10^ S/m respectively [74]. In contrast highly conductive materials like copper and graphite reach 10^6^ S/m and 10^4^ S/m [75]. Biological tissues fall in the intermediate range, for instance, with bone at about 10^−3^ S/m [76] and muscle near 10^0^ S/m [10,77,78]. For the CNF samples, no conductivity could be detected (Figure 4B). The GNP samples showed conductivity with increasing GNP concentration, starting at one sample out of three being conductive at a calculated concentration of 15 wt.%, which corresponds to a measured concentration of 12 wt.% in the thermogravimetric analysis. For all four samples with a calculated concentration of 20 wt.% GNPs, which corresponds to a measured concentration of 16 wt.%, a conductivity of 2.7 µS/m could be measured. This conductivity is an improvement over pure PLA, albeit still in the lower range of conductivity.

The sudden onset of conductivity can be explained by percolation theory, which describes the formation of a conductive path, i.e., the percolation network, through a non-conducting matrix (e.g., PLA) once the filler concentration exceeds a critical threshold [79]. Here, the conductivity does not increase linearly with filler concentration, but rather jumps at the critical percolation concentration, and eventually reaches a plateau at the conductivity level of the pure filler (Figure 4C) [80]. The critical concentration in our study was 15 wt.% (calc.) for GNP-containing filaments for some samples, though a reliable formation of the percolation network could be detected in samples with 20 wt.% (calc.) GNP.

Using the simple power-law model, the theoretical conductivity σ at a specific filler volume fraction $V_{f}$ above the percolation threshold $V_{C}$ can be described (Equation (2)) [81,82]. The model describes the exponential increase in conductivity at filler concentrations higher than the percolation threshold:

(2) $$\sigma =\sigma_{0}{\left(V_{f}-V_{C}\right)}^{S}$$

where $\sigma_{0}$ is the electrical conductivity of the filler and s is a conductivity exponent, which is influenced by particle shape, orientation, polymer–particle interaction and particle dispersion. A curve fitting and a reliable determination of the factor is not possible in this this study due to the low number of experimental data points. However, other studies on GNP-composite systems report experimentally determined values of s of 1.6–2 [79,81,82].

The percolation threshold $V_{C}$ is also influenced by the particle shape, orientation and particle dispersion. A key factor in the formation of the percolation network is the aspect ratio of surface area to volume of the filler particles [66]. With a high aspect ratio, the probability of contact between the particles increases and the required critical concentration decreases. The formation of the percolation network starts at a 5 nm particle distance due to the tunnel effect, in which electrons can jump over non-conductive distances [22,66,80,83]. Masarra et al. showed a similar percolation threshold on 15% GNP in a PLA/PCL matrix, achieving an electrical volume resistivity of 660 Ω cm. However, they observed an additional local increased concentration of particles in PCL nodules in the PLA, achieving a conductivity 10× higher than the one measured in this study [22]. Dul et al. determined their electrical percolation threshold at 7.3 wt.% for GNP particles and 0.9 wt.% for carbon nanotubes in acrylonitrile–butadiene–styrene (ABS), and therefore, observed a lower conductivity than the one in this study. Furthermore, this highlights the influence of particle shape. Still, this value was obtained with an electrical resistance of 50 kΩcm at 15 wt.% GNP, suggesting that the resistance measuring system was more sensitive than the one used in the present study [40]. Conductive hydrogel-based approaches like poly-pyrrole-doped collagen scaffolds feature an even higher conductivity of around 0.5–1.4 mS/m due to their matrix material, namely, the hydrogel, also being conductive [10,11].

Human tissues, like muscle and nerves, have a conductivity that is 10^5^ times higher (0.6–2.51 S/m) than the 20 wt.% (calc.) GNP filament [10,77,78]. Hence, when including 20 wt.% (calc.) GNP composite electrodes in tissue engineering applications, further constructive design could be applied to avoid creating a short circuit. In order to deliver voltages to a target area deep in the tissue, the system’s conductivity should be higher than the tissue it touches. For example, the muscle requires it to be higher than 0.6 S/m [77] to avoid a short circuit. Alternatively, the conductive 20 wt.% (calc.) GNP filaments developed during this study could be insulated with a surface layer of PLA without additives via fused filament fabrication to stimulate the targeted area.

For samples with a GNP content of 15 wt.% (calc.), the printing orientation, either parallel or perpendicular to the measurement direction, did not significantly influence the electrical resistivity in the present study (Figure 4D). This contrasts with previous reports indicating that the printing direction can introduce porosity and induce particle orientation due to shear forces between the molten composite and the inner nozzle wall, thereby altering the electrical conductivity [22,73]. However, looking at the cross-sectional SEM images (Figure 4E), only a little to no porosity is visible in the measurement direction (y), resulting in a mostly connected material, which does not lower the electrical conductivity. Interestingly, the porosity perpendicular to the measurement direction (x) is higher for samples printed in the y-direction, although it is still low at 1.7% (Figure 4F). However, these pores are not hindering the conductivity, as the printed paths are still connected. The discrepancies between the literature and our study highlight the importance of optimizing the printing parameters to minimize printing-induced porosity, which is particularly critical for filaments exhibiting low intrinsic conductivity.

For a qualitative demonstration of the manufactured filaments conductivity, all following experiments were done with the filament with the highest conductivity, namely, 20 wt.% (calc.) GNPs. For visualization, an LED was successfully driven as part of an electrical circuit using a voltage generator at 16 V and a 20 wt.% (calc.) GNP printed sample (Figure 4G). The latter was prepared beforehand by covering the contact points with conductive silver paint to increase the likelihood of contact with a conductive path in the percolation network, as the samples filler content is close to the onset of conductivity. Using the same setup with a 16 V direct current, the surface temperature of the printed sample was measured using an infrared thermal camera (Figure 4H). As expected, over the time span of 14 min, no significant heat production could be measured, as the electrical power was the product of the system’s voltage and current, which was very low due to the high resistance of the filament. Dul et al. also only achieved measurable thermal heating with highly conductive samples (>5 mS/m) [40]. The non-measurable temperature change is favorable for the application of developed composite filaments in TE, as mammalian cells cannot survive prolonged timespans with temperatures above 37 °C [84].

### 3.5. Biological Characterizations

**Figure 5:**
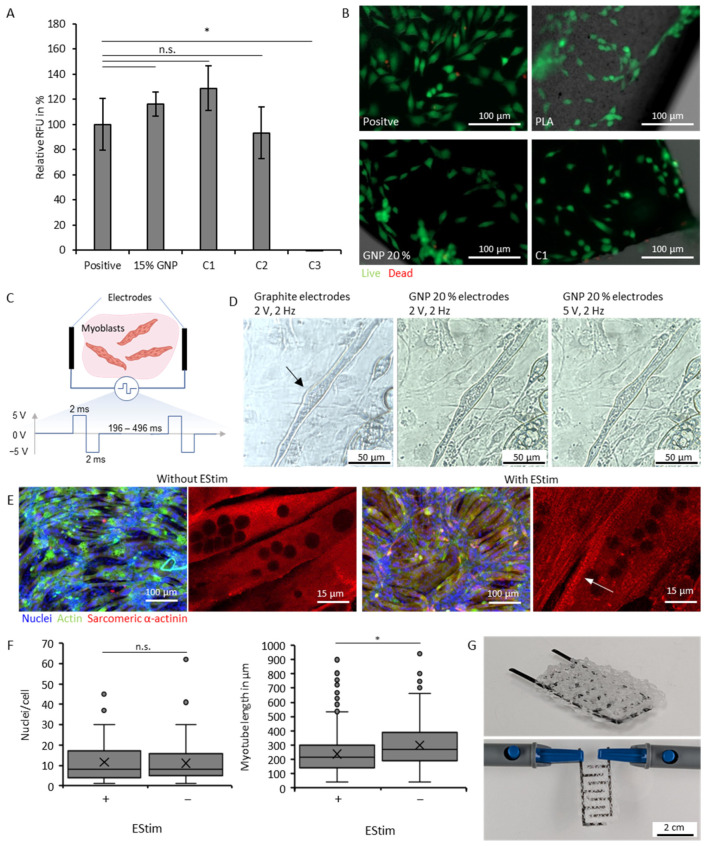
Biological characterization of the filaments and characterization of the usability of the filaments for electrical stimulation of C2C12 myoblast cells. (**A**) Cytocompatibility of different commercial filaments compared with the cultivation of cells without filaments. (**B**) Fluorescence microscopy image after one day of C2C12 cells seeded on top of printed samples and a well plate (green: live, red: dead). (**C**) Scheme of the electrical stimulation setup with a pulsed bi-phasic signal at 1–5 V and 2–5 Hz. (**D**) Microscopic image of a mature myotube contracting using electrical stimulation at 2 V and graphite electrodes (black arrow) and not contracting using printed 20 wt.% (calc.) GNP electrodes at 2 V and 5 V. (**E**) Fluorescence microscopic images of cells cultivated without and with continuous electrical stimulation of 4 days at 3 V (blue: nuclei, green: actin, red: sarcomeric α-actinin). (**F**) Quantification of cell morphology with and without electrical stimulation for 4 days. (**G**) FFF printed PCL scaffold for muscle tissue engineering with integrated printed electrodes from 20 wt.% GNP. Statistical significance * for *p* < 0.05. n.s. for no statistical significance.

Finally, we investigated the biological properties of the 20 wt.% (calc.) GNP filament for exemplary use in muscle tissue engineering (Figure 5). Cytocompatibility of the produced conductive filament was analyzed and compared with commercially available filaments using the myoblast cell line C2C12 (Figure 5A). The GNP samples achieved high cytocompatibility (117%), with a similar metabolic activity or cell count to the positive control, which was set to 100%.

The results of the three commercial substantially filaments differ, highlighting the importance of the filler materials, as well as additives for the cytocompatibility, since the matrix material of the C1 and C2 filaments is PLA with carbon black as their conductive filler, which are referred in the literature as cytocompatible; however, in long-term studies, the genotoxic effect of carbon black could become visible [37,61]. They achieve signals of 129% and 93% respectively. The C3 filament presents excellent conductivity (0.006 Ωcm) [37], but no metabolic activity could be measured, most likely due to its included conductive copper fillers. C1 and C2 filaments’ effects on cell viability are not significantly different to the positive control of the cells cultured under standard conditions in a well plate. Cells seeded on top of the printed samples of pure PLA, 20 wt.% (calc.) GNP, and C1 show good attachment, as well as a healthy elongated morphology (Figure 5B). The results from the cytocompatibility analysis (Figure 5A,B) are promising for use of the 20 wt.% (calc.) GNPs and the C1 filaments in tissue engineering applications. However, in order to make conclusive statements about biocompatibility, long-term in vitro and in vivo studies are required, as short-term studies are only of limited significance.

For the 20 wt.% (calc.) GNP filament, we also expect long-term biocompatibility when implanted in a patient due to PLA being commonly used in tissue engineering, with only the slight drawback of acidic degradation [85]. The exact composition of the C1 filament is not publicly available, so no statement can be made regarding the long-term effect; however, according to our mechanical tests, the C1 filament seems to include materials like plasticizers or extrusion aids, which can have long-term negative effects [86].

For in vivo use, the degradation rate of the printed composite filaments has to be considered. For scaffolds for bone regeneration, as well as muscle tissue electrostimulation, a supported maturation phase after 3–5 weeks is needed for the cells to form a tissue precursor. During this time span, the composite should be conductive and mechanically and geometrically stable. Afterwards, hydrolytic degradation is helpful to not hinder the newly formed tissue.

Initial degradation of the surface can increase the conductivity of the surrounding due to removal of the insulating non-conductive PLA matrix [87]. Later-stage bulk degradation and filler release reduce the conductivity.

The molecular weight, crystallinity and GNP content have an influence on the hydrolytic degradation rate. Here, several effects of the fillers come into effect. The fillers can increase the crystallinity of the composite [88], and in higher concentrations (>1 wt.%), they can act as a hydrophobic barrier, and therefore, lower the degradation rate [89]. While the molecular weight can be reduced by melt mixing [90], an imperfect binding of the GNPs with the PLA matrix can also act as a leading path for the hydrolytic solution, speeding up the degradation due to a better overall penetration of the scaffold [88].

For each use, the degradation rate of the composite has to be adjusted according to the scaffold geometry, macroscopic porosity and surface treatment, for instance, O_2_-plasma treatment to increase hydrophily [88].

Finally, we developed an experimental setup for investigating the biological functionality of the electrically conductive components (Figure 5C). A signal generator using an STM32 microcontroller, an amplifier, and signal filters with interchangeable electrodes produces a pulsed biphasic electrical signal in the range of 0–5 V at variable frequencies (0–2000 Hz). The electrodes can be sterilized and placed in a well plate to stimulate cells. The test setup allowed for the investigation of two different important influencing factors: the stimulation of already matured muscle cells to promote muscle contraction (Figure 5D) and an investigation to support cell differentiation, and thus, shorten the time needed for it (Figure 5E).

For electrical stimulation of C2C12 myoblasts, rectangular pulsed two-phase signals in the range of 1–5 V/cm and 1–10 Hz have already been described in literature [4,5,91,92]. We first validated the measurements with graphite electrodes, to establish the experimental setup. When applying electrical stimulation on already mature myotubes, its contraction can be achieved, improving the force generation, and therefore, functionality of the tissue [5]. For this, C2C12 cells were cultured in growth media for 3 days, and then for 3 weeks in differentiation media. Using the graphite electrodes, we were able to achieve synchronous contraction at 2 V/cm of 2D cell culture at the frequency of the signal (Figure 5D, Video S1). The resulting amperage using PBS as the surrounding liquid was 0.16 mA. When applying the same signal with 3D-printed 20 wt.% (calc.) GNP electrodes at 2 V and 5 V, no contractions were visible, suggesting that a significant part of the voltage is already lost at the electrodes due to their high electrical resistance. Hence, the voltage and amperage produced by the signal generator is too low due to the filaments’ low conductivity being unable to overcome the activation threshold of the cells. However, by altering the setup to allow for higher voltages, stimulation using the manufactured filaments could be possible.

To improve the myoblasts’ maturation, cells were cultured in differentiation media for four days until early myotubes appeared. After continuous electrical stimulation with the graphite electrodes for additional four days with the pulsed signal at 3 V/cm, differentiation was observed and analyzed (Figure 5E). In our study, the number of nuclei in each myotube was similar between the samples with and without electrical stimulation (Figure 5F). However, the myotube length was slightly increased for myotubes with electrical stimulation. Furthermore, electrostimulated samples showed the formation of z-stripes in the distribution of sarcomeric α-actinin, an indicator of mature myotubes.

Due to the setup’s adjustability regarding electrical signals and electrode materials, we consider the design to be useful for characterizing materials with higher conductivity than the ones produced in this study in terms of cell biology in the future regarding analyzing contractility and maturation duration. To achieve higher conductivity, a higher filler content in the filaments is needed. However, the filler content is limited by the achievable shear forces in the current setup. To increase the filler content of filaments made using the melt-mixing process, machines with a higher force output have to be used or the viscosity the molten polymer has to be lowered. For the latter, smaller molecular weights of the PLA could be used, with the drawback of altering the degradation time [52]. Alternatively, research has to be done in the area of cytocompatible plasticizers. Here, lignin and PCL were previously successfully used; however, they did not assess the cytocompatibility of their filaments [22,93,94].

Finally, we integrated the 20 wt.% (calc.) GNP filament into a scaffold designed for muscle tissue engineering in a combined 3D printing approach. The scaffold, which was based on our previous work, was modeled after a clinical case of a volumetric muscle loss [95]. The scaffold can be filled with a cell-laden hydrogel. The flexible porous PCL scaffold offers mechanical and geometrical cues, while a conducting filament could offer electrical cues for improved functionality of engineered muscle tissues. The generated tissue could be an alternative treatment for volumetric muscle loss in regenerative medicine.

Overall, this study showcases the possibilities of electrically conductive 3D-printable filaments for use in muscle tissue engineering scaffolds. It also highlights the necessary considerations of the material choice and production processes.

## 4. Summary and Conclusions

Electrical stimulation is a way to support functionality in biofabricated tissue to achieve improved usability and reduced production times. Several tissues benefit from electrical stimulation, especially muscle tissue for implants in regenerative medicine.

Scaffolds from conductive 3D-printable filaments can be shaped to each patient’s size and allow for the locally defined application of electrical signals, as well as the inclusion of additional maturation, promoting stimuli like pore sizes or fibrous structure to support cell alignment by contact guidance [3,7].

Current commercially available conductive filaments usually use a non-degradable base material and additives such as plasticizers, lubricants, and thermal stabilizers to improve the manufacturing and printability [36,37]. These additives can negatively affect the viability and biofunctionality of cultured cells [38].

For the improvement of muscle maturation and following the implantation of biocompatible conductive filaments without production aids and additives, PLA-based filaments with electrically conductive fillers of GNPs and CNFs for electrical functionalization were developed.

A two-stage process using an additional twin-screw extruder to the single-screw extrusion was preferred due to the higher amounts of fillers that could be incorporated in the filaments. The maximum filler content that could be homogeneously integrated in the PLA matrix using this setup was 3.6 wt.% CNF and 16 wt.% GNP, as measured with TGA. The measured contents were lower than the calculated values (5% for CNF and 20% for GNP) by 1.4% and 4%, respectively.

Using all filaments made in the two-stage process, it was possible to print geometries with a direct drive extruder that were successfully shown to aid cell alignment (around 400–600 µm) [3,7,8,9]. It was necessary to optimize the printing parameters (e.g., printing temperature, extrusion factor, printing speed).

When analyzing the CNF-filled filaments, no conductivity could be detected using a four-probe setup. The critical concentration threshold for the conductive GNP particles to form a robust conductive percolation network was 16 wt.% (measured) with conductivity of 2.7 µS/m. The literature describes a similar onset of conductivity for the same filler concentrations [22,40]. Using a printed sample from the 20 wt.% (calc.) GNP filament, we were able to drive an LED successfully as a part of an electrical circuit using a voltage generator at 16 V.

The 20 wt.% (calc.) GNPs was cytocompatible when looking at myoblast viability and cell morphology seeded on top of printed samples. The cells showed good attachment and a healthy elongated cell morphology. Also, two commercially available PLA-based filaments using carbon black as conductive filler (C1, C2) achieved a cell viability similar to the control as well, while the filament using copper as a conductive filler (C3) failed in the cytocompatibility test, with no cells surviving.

Finally, we introduced an adjustable electrical setup regarding electrical signals and electrode materials, which can be useful for characterizing other materials with higher conductivity in relation to cellular responses in the future. With the setup and commercially available graphite electrodes we were able to show the improvement of muscle maturation and the triggering of contractions of muscle cells.

## Figures and Tables

**Table 1:** Compounding parameters of the twin-screw extruder.

Compound	T1 (Nozzle) in °C	T2 in °C	T3 in °C	T4 in °C	T5 in °C	T6 in °C	T7 in °C	T8 in °C	Screw Speed in rpm	Torque in %
GNP	200	195	190	190	185	185	180	170	350	55
CNF	210	205	200	195	190	185	180	170	250	38

**Table 2:** Extruding parameters of the single-screw extruder.

T1 (Nozzle) in °C	T2 in °C	T3 in °C	T4 in °C	T5 in °C	Screw Speed in rpm	Fan Speed in %
165	183	180	160	130	350	55

**Table 3:** SEM and SEM EDX parameters.

	Measurement	Voltage in kV	Current in µA	Pressure in Pa	Magnification
Cryofracture of filament	EDX	10	100	1.4 × 10^−4^	623×
Tensile fracture of filament	SEM	10	100	1.4 × 10^−4^	100×, 500×
Fracture of printed parts	SEM	10	100	1.5 × 10^−4^	495×, 500×

## Data Availability

The original contributions presented in this study are included in the article/Supplementary Materials. Further inquiries can be directed to the corresponding authors.

## Supplementary Materials

The following supporting information can be downloaded from https://www.mdpi.com/article/10.3390/polym18091058/s1, Video S1: Microscopy of electrical stimulation with a pulsed bi-phasic signal at 2–5 Hz of a mature myotube contracting using the electrical stimulation at 2 V and graphite electrodes (black arrow) and not contracting using printed 20 wt.% (calc.) GNP electrodes at 2 V and 5 V.

## Abbreviations

The following abbreviations are used in this manuscript:

Calc.	calculated
GNP	graphene nanoplatelet
CNF	carbon nanofiber
Wt.	weight
PLA	polylactic acid
PCL	polycaprolactone
TGA	thermogravimetric analysis
DTGA	derivative thermogravimetric analysis
CAD	computer-aided design
